# Association of *CETP TaqI *and *APOE *polymorphisms with type II diabetes mellitus in North Indians: a case control study

**DOI:** 10.1186/1472-6823-5-7

**Published:** 2005-07-01

**Authors:** Manjusha Dixit, Sandeep Bhattacharya, Balraj Mittal

**Affiliations:** 1 Department of Genetics, Sanjay Gandhi Postgraduate Institute of Medical Sciences, Lucknow-226014 India

## Abstract

**Background:**

Genetic variants of proteins involved in lipid metabolism may play an important role in determining the susceptibility for complications associated with type II diabetes mellitus (T2DM). Goal of the present study was to determine the association of cholesteryl ester transfer protein *Taq*I B, D442G, and *APOE Hha *I polymorphisms with T2DM and its complications.

**Methods:**

Study subjects were 136 patients and 264 healthy controls. All polymorphisms were detected using PCR-RFLP and statistical analysis done with χ^2 ^test and ANOVA.

**Results:**

Although *CETP Taq*I B polymorphism was not associated with the T2DM, yet B1B2 genotype was significantly (p = 0.028) associated with high risk of hypertension in diabetic patients (OR = 3.068, 95% CI 1.183–7.958). In North Indians D442G variation in *CETP *gene was found to be absent. Frequency of *APOE HhaI *polymorphism was also not different between patients and controls. In diabetic patients having neuropathy and retinopathy significantly different levels of total-cholesterol [(p = 0.001) and (p = 0.029) respectively] and LDL-cholesterol [(p = 0.001) and (p = 0.001) respectively] were observed when compared to patients with T2DM only. However, lipid levels did not show any correlation with the *CETP Taq*I B and *APOE Hha *I genetic polymorphisms.

**Conclusion:**

*CETP Taq*I B and *APOE Hha*I polymorphism may not be associated with type II diabetes mellitus in North Indian population, however *CETP Taq*I B polymorphism may be associated with hypertension along with T2DM.

## Background

Dyslipidemia is a major cardiovascular risk factor in type II diabetes mellitus (T2DM) with coronary heart disease being the most common cause of death. Risk relates to raised triglycerides (TG) and decreased high density lipoproteins (HDL) as well as raised low density lipoproteins (LDL). In diabetes, lipid risk thresholds are lower and interactions with other cardiovascular risk factors are more powerful, compared with general population. Hypertension is upto twice as common in diabetic patients as in general population [1]. Studies have shown that lipid abnormalities might contribute to the development and progression of diabetic nephropathy [2]. Hypercholesterolemia is a major determinant of decline of renal function in patients with diabetes [3].

Genetic polymorphisms of the enzymes and proteins involved in lipid metabolism like cholesteryl ester transfer protein (CETP) and apolipoprotein E have been shown to affect plasma lipid concentrations [4]. CETP modifies HDL, LDL and very low density lipoprotein (VLDL) levels. It transfers cholesterol esters (CE) from CE rich particles (HDL and LDL) to triglyceride rich particles (VLDL) in exchange of triglyceride from the latter [5]. There has been an ongoing debate as to whether CETP is pro- or anti-atherogenic as it provides a mechanism for the transfer of cholesterol from the cardioprotective HDL subfraction to the potentially atherogenic LDL subfraction [6]. *CETP *gene encompasses 16 exons and it has been localized on chromosome 16q21. Several genetic polymorphisms have been reported which may be associated with alteration in CETP activity. *Taq*I B polymorphism has been most widely studied, which results from a silent mutation in nucleotide 277, in intron 1 of the gene. The polymorphism has been associated with decreased CETP mass and an increase in HDL-cholesterol [7-9]. The *Taq*I polymorphism B1 allele of *CETP *has been shown to be an independent risk factor for development of cerebral vascular disease, in patients with T2DM [10]. The locus has also been reported to modulate the risk for diabetic complications in patients with T2DM and effect seems to be different between men and women [11]. Another polymorphism D442G (Asp442-->Gly) in exon 15 of *CETP *is located close to the active site of the enzyme and leads to reduced plasma CETP mass and specific activity [12]. The mutation is more prevalent in Japanese subjects with high HDL-levels (>100 mg/dL) [12-14].

Apolipoprotein E (Apo E) plays a central role in clearance of lipoprotein remnants by serving as a ligand for LDL and apo E receptors. The gene for apo E is approximately 3.7 kb in length and contains 4 exons. It has been mapped to chromosome 19q13 in humans. Three different *APOE *alleles (ε2, ε3, and ε4) give six phenotypes. The protein isoforms result from interchanges of cysteine and arginine at the residue 112 and 158. In apo E2, cysteine occurs at both positions; in apo E4 arginine is located at both positions; and in apo E3 cysteine occupies position 112 and arginine, position 158. Based on homozygosity or heterozygosity of these apo E isoproteins, a total of six phenotypes (E2/2, E3/3, E4/4, E4/2, E4/3, and E3/2) are present in the population [15]. *APOE *gene polymorphism has been shown to be associated with the development of diabetic nephropathy in T2DM patients in Taiwan [2]. Apo ε4 allele may speed up the rate of decline of glomerular filtration rate (GFR) in patients with progressive diabetic renal disease [16]. Studies have shown ε2 as positive and ε4 as negative factor for the progression of diabetic nephropathy [17,18].

Genetic epidemiologic studies, familial aggregation, familial transmission pattern, and twin concordance rates suggest the importance of genetic susceptibility underlying the development of T2DM. High incidence of disease in North Indian population and lack of study exploring the genetic basis of diabetes made us to study the association of *CETP Taq*I B and D442G; and *APOE Hha*I polymorphisms with T2DM.

## Methods

### Subjects

The study comprised of 136 T2DM patients (mean age 46.96 ± 9.38 yr.) and 264 healthy individuals (mean age 47.39 ± 16.64 yr.). Patients were enrolled from the outpatients attending the clinics of Sanjay Gandhi Postgraduate Institute of Medical Sciences from November 2003 to April 2004. Most patients belonged to State of Uttar Pradesh in North India. Subjects were classified as diabetic if they had fasting glucose concentrations ≥126 mg/dL or 2-hour glucose concentrations ≥200 mg/dL after a 75-g oral glucose tolerance test [19,20]. These patients were further classified according to their complication into groups – neuropathy, nephropathy, retinopathy, hypertension, and T2DM without complication, after reviewing their medical chart. Neuropathy was defined as presence of distal sensory loss in feet. Nephropathy was defined as presence of gross albumin in urine >300 mg/d in 24 hr collection of urine sample. Retinopathy was defined as presence of retinal vascular microaneurysm, blot hemorrhages, or cotton wool spots. Hypertension was defined as presence of blood pressure of ≥140/90 mm Hg at three different readings on different days. None of the patients were on lipid lowering therapy at the time of drawing their sample. All the patients were on oral hypoglycemic agent Glimepride or Metformin or both.

The controls were the healthy staff members of institute with negative oral glucose tolerance test. Only those patients and controls were included who did not have the history of coronary artery disease, neoplasia, or other metabolic disorder.

Study was approved from the ethical committee of the institute. After an informed consent an overnight fasting blood sample was taken in EDTA for analysis of lipids and DNA. The plasma was separated by centrifugation at 3000 rpm within 10 minutes of blood collection for the lipid analysis. The genomic DNA was extracted from peripheral blood leucocytes pellet using the standard salting out method [21].

### Lipids levels

In plasma, total cholesterol, triglyceride, and HDL-cholesterol were analyzed using commercially available kits (Accurex Biomedical Pvt. Ltd., Mumbai, India). For HDL-cholesterol estimation, selective precipitation of other lipoproteins was done using sodium tungstate and magnesium chloride. LDL-cholesterol was calculated according to previously described method [22].

### Genotyping

#### *CETP Taq*I B and D442G polymorphisms

A fragment of 535 bp in intron 1 of the *CETP *gene was amplified by polymerase chain reaction (PCR) in a DNA thermal cycler (DNA Engine PTC-100, MJ Research, Inc) using primers forward 5'-CACTAGCCCAGAGAGA-GGAGTGCC-3' and, reverse 5'-CTGAGCCCAGCCGCACACTAAC-3' [23]. For D442G polymorphism analysis primers used to amplify exon 15 and flanking sequences were as follows: forward 5'-GTGTTTACAGCCCTCATGAAC-3' and reverse 5'-AAGCCAAAGTCCATCTCTGCAG-3' [24]. Each amplification was performed using 200 ng of genomic DNA in a volume of 25 μl using 12.5 pmol of each primer, 200 μM each dNTPs, 15 mM MgCl_2_, 100 mM Tris and two units of *Taq *polymerase (Bangalore Genei, Bangalore). DNA templates were initially denatured at 95°C for three minutes, followed by 30 cycles with denaturation at 95°C for 30 seconds, annealing at 60°C for 30 seconds and, extension at 72°C for 45 seconds and finally, an extension at 72°C for five minutes. The PCR products were subjected to restriction digestion with 4 U of the restriction endonuclease *Ta*qI for 15 μl of PCR sample at 65°C for three hours in the buffer recommended by the manufacturer (MBI Fermentas, USA).

*Taq*I B genotype was determined by electrophoresis of restricted PCR product on 2% agarose gel followed by ethidium bromide staining. Presence of *Taq*I site (B1 allele) gave two bands of 174 bp and 361 bp and absence (B2 allele) showed one band of 535 bp.

For genotyping of D442G polymorphism, *Taq*I digested PCR products were run on 20% polyacrylamide gel. This product has two sites for *Taq*I. Homozygous subjects with DD give two bands (218 bp and 69 bp) and heterozygous show four bands (218 bp, 69 bp, 41 bp and 28 bp) and homozygous for G allele show three bands (218 bp, 41 bp, and 28 bp).

#### *APOE *polymorphism

The primers used to analyze the *APOE *were as follows: Forward 5'-ACAGAATTCGCCCCGGCCTGGTACAC-3' and Reverse 5' TAAGCTTGGCACGGCTGTCCAAGG A-3' [25]. The PCR products were initially denatured at 95°C for five minute, cycling conditions were 95°C for one minute, 58°C for one minute, 70°C for one minute (30 cycles) followed by final extension at 72°C for 10 minutes. After PCR amplification, 10 units of *Hha*I were added for digestion (>3 h at 37°C). The digested product was run on 20% polyacrylamide gel at 300 V, stained with ethidium bromide and visualized under ultraviolet light.

### Statistical evaluation

Data was analyzed using the statistical software (SPSS vs.11.5). Direct gene counting method was used to determine the frequency of genotypes and alleles. The chi-square test or Fisher's exact test was used to determine differences in frequencies. *P*-value < 0.05 was considered as significant. All continuous variables were expressed as mean ± SD. The normality in the distribution of total-cholesterol, HDL-cholesterol, triglyceride and LDL-cholesterol was confirmed by using normal probability plots. Since total-cholesterol, HDL-cholesterol, triglyceride and LDL-cholesterol were not distributed normally they were naturally log transformed. Significant covariates for each dependent trait were identified using Pearson's correlation with 5% level of significance. Sex and BMI were found to be significant covariates for total cholesterol, sex for triglyceride and HDL-cholesterol, and BMI for LDL-cholesterol. Total cholesterol, triglyceride, HDL-cholesterol, and LDL-cholesterol values were subjected to a linear regression procedure to obtain adjusted values for significant covariates. ANOVA was performed to determine genetic source of variation for biochemical traits in control population separately. Lipid variables were taken as dependent variable and the genetic marker as independent. If this test was significant, Tukey posthoc test was performed to find out which genotype/allele differed significantly from others. All calculations were done in total samples as well as in males and females separately

## Results

Table 1 and table 2 show the demographic and lipid profile of study population respectively. Plasma concentrations of total-cholesterol (*p *= 0.001) and LDL-cholesterol (*p *= 0.00008) levels were significantly higher in T2DM patients than in controls but HDL-cholesterol and triglyceride levels were not significantly different (Table 2 – unadjusted values). After stratification of population on the basis of gender same pattern of lipid profile was found. Overall, males had lower circulating levels than females in patients as well as in controls.

**Table 1:** Demographic profile of T2DM patients and controls

	**Patient**			**Control**		
	Total	Male	Female	Total	Male	Female

**Age (year)**	46.96 ± 9.38	48.17 ± 10.09	45.32 ± 8.13	47.37 ± 16.66	50.76 ± 19.81	44.71 ± 13.15
**Height (cm)**	160.33 ± 7.74	164.20 ± 6.80	153.43 ± 3.19	161.21 ± 6.65	166.29 ± 6.47	157.57 ± 3.82
**Weight (kg)**	62.75 ± 12.11	64.83 ± 11.76	59.04 ± 12.08	60.86 ± 9.26	64.76 ± 9.64	57.98 ± 7.90
**BMI (kg/m^2^)**	24.37 ± 4.21	23.98 ± 3.73	25.07 ± 4.97	23.34 ± 2.68	23.41 ± 2.81	23.29 ± 2.61

Values in mean ± SD

**Table 2:** Total cholesterol, triglyceride, HDL-cholesterol and LDL-cholesterol concentrations in T2DM patients and controls

	**Patients^#^**			**Control^#^**		
	Total (n = 83)	Male (n = 48)	Female (n = 35)	Total (n = 135)	Male (n = 87)	Female (n = 48)

Total-Cholesterol*◆ (mg/dl)	**187.24 (53.43)**	**179.13 (53.18)**	**198.37 (52.49)**	**150.22 (41.18)**	**143.42 (28.24)**	**162.44 (56.02)**
Triglyceride* (mg/dl)	186.52 (92.22)	172.52 (87.09)	205.71 (96.81)	145.67 (50.56)	138.50 (41.43)	158.68 (62.29)
HDL Cholesterol* (mg/dl)	39.61 (6.54)	37.63 (5.97)	42.34 (6.36)	38.79 (6.77)	38.05 (6.99)	40.12 (6.19)
LDL-cholesterol*◆ (mg/dl)	**110.32 (46.84)**	**107.00 (47.46)**	**114.89 (46.25)**	**82.30 (38.58)**	**77.72 (26.42)**	**90.59 (53.45)**

Values: (mean ± SD)

# Lipid profile was available in 83 T2DM patients and 135 controls

* Unadjusted values have been shown

◆ Significant value

### *CETP Taq*I B polymorphism

In controls the frequency of B1 and B2 alleles of *CETP Taq*I B gene was 52.65% and 47.35% respectively, which is similar to most of Caucasian populations. The observed genotypes B1B1, B1B2, and B2B2 were in Hardy Weinberg equilibrium. Analysis of the polymorphism showed that the frequency of B2 allele and B2B2 genotype was not significantly different in patients and controls (43.75% vs 47.35% and 16.91% vs 21.97%, Table 3). Further stratification of data according to gender (Table 4) did not show any association with the disease. After subdividing study population into different age groups no significant association was found but B1B2 genotype showed weak association and risk (*p *= 0.054; OR 3.826, 95% CI 0.916–15.98) for disease in 61–75 age group (Table 5).

**Table 3:** *CETP Taq*I B and D442G polymorphisms: allele and genotype frequencies in T2DM patients and controls

	Patient* n (%)	Control* n (%)	***P*-value**	**OR (95% CI)**
**B1B1**	40 (29.41)	72 (27.27)	0.652	1.11 (0.70–1.76)
**B1B2**	73 (53.68)	134 (50.76)	0.580	1.12 (0.74–1.70)
**B2B2**	23 (16.91)	58 (21.97)	0.233	0.72 (0.42–1.23)
**B1**	153 (56.25)	278 (52.65)	0.333	1.16 (0.86–1.55)
**B2**	119 (43.75)	250 (47.35)	0.333	0.87 (0.64–1.16)
**DD**	136 (100)	264 (100)	-	-
**GG**	0 (0)	0 (0)	-	-

* Total T2DM patients 136 and controls 264

**Table 4:** Allele and genotype frequencies of *CETP Taq*I B polymorphism in T2DM patients and controls after stratifying in male and female

	Male*				Female*			
	Patient n (%)	Control n (%)	*P*-value	OR (95% CI)	Patient n (%)	Control n (%)	*P*-value	OR (95% CI)

**B1B1**	22 (27.8)	34 (29.3)	0.825	0.93 (0.49–1.76)	18 (31.6)	38 (25.7)	0.395	1.34 (0.68–2.61)
**B1B2**	44 (55.7)	60 (51.7)	0.585	1.17 (0.66–2.08)	29 (50.9)	74 (50.0)	0.910	1.04 (0.56–1.91)
**B2B2**	13 (16.5)	22 (19.0)	0.654	0.84 (0.40–1.79)	10 (17.5)	36 (24.3)	0.297	0.66 (0.30–1.44)
**B1**	88 (55.70)	128 (55.17)	0.920	1.02 (0.68–1.53)	65 (57.02)	150 (50.68)	0.249	1.29 (0.84–2.00)
**B2**	70 (44.30)	104 (44.83)	0.920	0.98 (0.65–1.47)	49 (42.98)	146 (49.32)	0.249	0.77 (0.50–1.20)

* Total male patients 79, controls 116 and total female patients 57, controls 148

**Table 5:** *CETP Taq*I B frequencies in T2DM patients and controls after subdividing according to age

**GENOTYPE**	**AGE (YEAR)** **(PATIENT NO./CONTROL NO.)**	***P*-VALUE**	**OR (95% CI)**
**B1B1**	Upto 30 (2/12)	0.599	2.000 (0.298–13.435)
	31–45 (15/23)	0.836	0.923 (0.430–1.980)
	46–60 (21/18)	0.186	1.650 (0.783–3.479)
	Above 60 (2/19)	0.481	0.433 (0.085–2.206)

**B1B2**	Upto 30 (3/28)	1.000	1.071 (0.164–7.014)
	31–45 (34/41)	0.243	1.508 (0.756–3.009)
	46–60 (27/40)	0.288	0.694 (0.354–1.363)
	Above 60 (8/23)	**0.054**	**3.826 (0.916–15.984)**

**B2B2**	Upto 30 (0/8)	1.000	-
	31–45 (7/17)	0.199	0.538 (0.207–1.398)
	46–60 (14/18)	0.879	0.940 (0.424–2.084)
	Above 60 (1/14)	0.433	0.300 (0.035–2.557)

When patient population was sub-divided according to the associated complications viz., neuropathy, nephropathy, retinopathy, hypertension, and no complication, B1B2 genotype was significantly (*p *= 0.018) associated with high risk of hypertension in diabetic patients (OR = 3.068, 95% CI 1.183–7.958), and B1B1 was found to be significantly protective (p = 0.038, OR = 0.139, 95% CI 0.018–1.093) for hypertension (Table 6).

**Table 6:** *Taq*I B *CETP *genotypes in various complications of T2DM

**CLINICAL COMPLICATION (NO. OF PATIENTS)**	**GENOTYPE (T2DM PATIENT NO. WITH COMPLICATION/WITHOUT COMPLICATION)**	**P-VALUE**	**OR (95% CI)**
**Nephropathy (29)**	B1B1 (8/24)	0.891	0.937 (0.365–2.403)
	B1B2 (15/42)	0.917	1.046 (0.449–2.437)
	B2B2 (6/17)	0.981	1.013(0.356–2.879)

**Neuropathy (12)**	B1B1 (4/24)	0.744	1.229 (0.338–4.468)
	B1B2 (5/42)	0.563	0.697 (0.205–2.375)
	B2B2 (3/17)	0.712	1.294 (0.316–5.307)

**Retinopathy (9)**	B1B1 (3/24)	0.719	1.229 (0.284–5.319)
	B1B2 (4/42)	1.000	0.781 (0.196–3.115)
	B2B2(2/17)	1.000	1.109 (0.211–5.830)

**Hypertension (29)**	B1B1 (1/17)	**0.038**	**0.139 (0.018–1.093)**
	B1B2 (22/42)	**0.018**	**3.068 (1.183–7.958)**
	B2B2 (6/24)	0.389	0.641 (0.232–1.772)

### *CETP *D442G polymorphism

For D442G no polymorphism was found in patient or control population, only D allele was present (Table 3).

### *APOE Hha*I polymorphism

Frequencies of all *APOE *genotypes were not significantly different between the diabetic and control group in total or after stratifying according to gender or age (Table 7).

**Table 7:** Apolipoprotein E gene polymorphism genotype and allele frequencies in T2DM patients and controls

	**Total^#^**		**Male^#^**		**Female^#^**	
	Patient n (%)	Control n (%)	Patient n (%)	Control n (%)	Patient n (%)	Control n (%)

E2E2*	1 (0.7)	0 (0)	1 (1.3)	0 (0)	0 (0)	0 (0)
E3E3*	101 (74.3)	197 (74.6)	55 (69.6)	88 (75.9)	46 (80.7)	109 (73.6)
E4E4*	1 (0.7)	1 (0.4)	1 (1.3)	0 (0)	0 (0)	1 (0.7)
E2E3*	11 (8.1)	19 (7.2)	8 (10.1)	9 (7.8)	3 (5.3)	10 (6.8)
E2E4*	4 (2.9)	6 (2.3)	2 (2.5)	5 (4.3)	2 (3.5)	1 (0.7)
E3E4*	18 (13.2)	41 (15.5)	12 (15.2)	14 (12.1)	6 (10.5)	27 (18.2)
E2*	17 (6.3)	25 (4.7)	12 (7.59)	14 (6.03)	5 (4.39)	11 (3.72)
E3*	231 (84.9)	454 (86.0)	130 (82.28)	199 (85.78)	101 (88.60)	255 (86.15)
E4*	24 (8.8)	49 (9.3)	16 (10.13)	19 (8.19)	8 (7.02)	30 (10.14)

*No significant difference between patients and controls

^# ^Total patients 136, 79 male and 57 female; and total controls 264, 116 male and 148 female

### Genetic polymorphisms and lipid profile

In diabetic patients having neuropathy significantly different levels of total cholesterol (p = 0.001) and LDL-cholesterol (p = 0.001) were found than in T2DM only patients. Unadjusted values were higher in T2DM patients with neuropathy than in patients with T2DM only (total-cholesterol, 225.89 ± 45.18 mg/dL vs. 186.45 ± 51.56 mg/dL and LDL-cholesterol, 152.33 ± 41.07 mg/dL vs. 105.72 ± 44.09 mg/dL respectively) but adjusted values were higher in patients with T2DM only than in patients with T2DM and neuropathy (total-cholesterol, 5.09 ± 0.07 vs. 5.00 ± 0.06 mg/dL and LDL-cholesterol, 4.45 ± 0.09 vs. 4.32 ± 0.11 mg/dL respectively). In T2DM patients with retinopathy adjusted total-cholesterol and LDL-cholesterol levels were significantly (p = 0.029 and p = 0.001 respectively) lower (5.00 ± 0.10 vs. 5.09 ± 0.07 mg/dL and 4.29 ± 0.11 vs. 4.45 ± 0.09 mg/dL respectively) than patients with T2DM only.

Lipid profile analysis did not show any significant difference in distribution among genotypes of *CETP Taq*I B polymorphism or *APOE Hha*I polymorphism. In T2DM patients though the levels of total-cholesterol and triglyceride were higher in ε4 allele carriers than in ε2 or ε3 allele carriers, but the difference was not statistically significant (Table 8).

**Table 8:** Lipid profile stratified according to *APOE *alleles

**Patient/Control**	**Lipid profile**	**ε2**	**ε3**	**ε4**
**Patients**	**Total-cholesterol (mg/dl)**	191.33 ± 60.98	185.69 ± 53.14	200.50 ± 51.47
	**HDL-cholesterol (mg/dl)**	38.44 ± 6.56	39.70 ± 6.60	39.50 ± 6.00
	**Triglyceride (mg/dl)**	143.22 ± 75.65	189.57 ± 93.10	183.14 ± 87.58
	**LDL-cholesterol (mg/dl)**	124.24 ± 48.92	108.07 ± 46.94	124.37 ± 41.61

**Controls**	**Total-cholesterol (mg/dl)**	172.55 ± 65.76	148.85 ± 39.45	147.28 ± 28.83
	**HDL-cholesterol (mg/dl)**	40.07 ± 5.19	38.73 ± 6.82	38.38 ± 7.26
	**Triglyceride (mg/dl)**	130.97 ± 32.69	145.93 ± 51.77	154.71 ± 46.61
	**LDL-cholesterol (mg/dl)**	106.29 ± 63.21	80.93 ± 36.58	77.96 ± 27.46

* Unadjusted values

## Discussion

To our knowledge, this is the first study of its kind in North Indian population. We studied association of *CETP Taq*I B, *CETP *D442G, and *APO*E *Hha *I polymorphisms with T2DM and with complications associated with the disease. In the present study, no association of *CETP Taq*I B polymorphism with the disease was found but B1B2 genotype was risk factor for hypertension along with diabetes (*P *= 0.028). However, B1B1 was found to be protective (*P *= 0.038).

CETP is a key regulator of lipid metabolism and polymorphism of the gene may be associated with complications of diabetes mellitus. B1B1 carriers have been reported to show lowest and B2B2 carriers highest HDL-cholesterol concentration [26-31]. but no association of *Taq*I B polymorphism was found in several other studies [32-34] A recent study in Singapore population comprising Chinese, Malays, and Asian Indians also showed that B2 allele was associated with high HDL-cholesterol concentration. Inspite of highest frequency of B2 allele, the HDL-cholesterol levels were lower in Asian Indians than in Chinese and Malays [35].

We found that B1B1 is protective for diabetes associated hypertension which seems contradictory to reports relating B1B1 with low HDL-cholesterol and B2B2 with high HDL-cholesterol. Relvas et al. [36] also found a higher prevalence of the B2B2 genotype of the CETP gene among diabetics than that observed in non-diabetics. These contradictory results indicate towards two possibilities. Firstly, *Taq*I B polymorphism is not the only determinant of HDL-cholesterol level, other polymorphisms or mutation in the *CETP *are more potent determinant. Various mutations/polymorphisms resulting in amino acid substitutions, namely A373P, I405V and R451Q, have been found to be independent variations from the *Taq*I B polymorphism effecting HDL-cholesterol and CETP activity [27,33,37-39]. Secondly, actual gene responsible/protective for hypertension may be different which is in linkage disequilibrium with *CETP Taq*I B polymorphism. Klerkx et al. [5] also showed that the *Taq*I B polymorphism is not instrumental in determining CETP or HDL-C levels, but is a marker for the -629 promoter variant.

D442G polymorphism has been implicated in alteration of CETP activity. The D442G acts in dominant negative manner with severe effect in heterozygous state [14]. A high prevalence of two different *CETP *gene mutations (D442G, 5.1%; intron 14G:A, 0.5%), was found in men of Japanese ancestry in the Honolulu Heart Program and mutations were associated with decreased CETP (-35%) and increased HDL-cholesterol levels (+10% for D442G) [40]. In our population exonic mutation D442G of CETP gene was not observed. In other populations of the world frequency of D442G varies greatly [41,42], in some it is not even polymorphic [43] (Table 9). In Japan, D442G mutation in CETP gene is around 7% in random male samples [44]. Frequency of the D442G substitution in Chinese showed enormous variations (0.2%–5%) [45-48] which may be due to ethnic differences in the studied populations. Absence of D442G polymorphism shows similarity of our population with Caucasians.

**Table 9:** Allele frequencies of *CETP *D442G polymorphism in various populations

**Population**	**D (%)**	**G (%)**	**Reference**
**Japan**	96.8	3.2	24
**Japan**	94.9	5.1	40
**Taiwan**	97.7	2.3	57
**Taiwan**	95.54	4.46	58
**Korea**	94.7	5.3	42
**Korea**	94	6	41
**China**	99.3	0.7	48
**China**	95	5	47
**China**	96.52	3.48	46
**China**	97.9	2.1	45
**UK**	100	0	43
**India**	100	0	Present Study

Earlier studies in T2DM patients have shown that ε2 and ε4 alleles of *APOE *are associated with high risk for dyslipidemia, nephropathy, retinopathy, and coronary artery disease [18,49-51] but other studies have shown contradictory results. Powell et al. [52] showed that *APOE *gene polymorphism is not linked to amyloid formation or progression of islet dysfunction in T2DM. No significant difference was found in *APOE *genotype frequencies between hypertriglyceridemic and normotriglyceridemic among T2DM patients [53]. Study in San Luis Valley, Colorado also showed, no significant effect of the *APOE *polymorphism on cholesterol levels among diabetics [54]. Another study in individuals with family history of diabetes showed that *APOE *polymorphism is not associated with lipids in men or women [55]. A previous study from Mumbai, India showed that *APOE *phenotype frequencies were not different between diabetic patients and healthy controls [56]. Our study also supports no association of *APOE Hha*I polymorphism with either T2DM or lipid variation. However molecular mechanism leading to lipid variation is not fully clear and various gene-gene and gene-environment interactions have also been observed, suggesting complex mechanisms leading to complications in T2DM patients.

Although our study found abnormal lipid profile in diabetes patients and its association with complications like diabetic neuropathy and retinopathy, yet these lipid profile abnormalities did not correlate with *CETP Taq*I B or *APOE Hha*I polymorphisms. In T2DM patients with neuropathy, unadjusted values of total-cholesterol and LDL-cholesterol were higher than, patients with T2DM only. After adjusting for covariates, results were opposite which shows the effect of covariates on lipid variation. The absence of any association of lipid profile with genetic subgroups suggest involvement of other environmental and genetic factors in the regulation of circulating lipid levels as well as the complex mechanism of T2DM.

## Conclusion

*CETP Taq*I B and *APOE Hha*I polymorphism may not be associated with type II diabetes mellitus in north Indian population, however *CETP Taq*I B polymorphism may be associated with certain complications along with T2DM.

## Competing interests

The author(s) declare that they have no competing interests.

## Authors' contributions

MD carried out the experimentation and data analysis. SB participated in clinical characterization of patients and experimentation. BM participated in the design of the study and coordination.

## Pre-publication history

The pre-publication history for this paper can be accessed here:


